# Defects of Protein Phosphatase 2A Causes Corticosteroid Insensitivity in Severe Asthma

**DOI:** 10.1371/journal.pone.0027627

**Published:** 2011-12-19

**Authors:** Yoshiki Kobayashi, Nicolas Mercado, Peter J. Barnes, Kazuhiro Ito

**Affiliations:** Airway Disease Section, National Heart and Lung Institute, Imperial College London, London, United Kingdom; University of Tübingen, Germany

## Abstract

**Background:**

Corticosteroid insensitivity is a major barrier of treatment for some chronic inflammatory diseases, such as severe asthma, but the molecular mechanism of the insensitivity has not been fully elucidated. The object of this study is to investigate the role of protein phosphate 2A (PP2A), a serine/threonine phosphatase, on corticosteroid sensitivity in severe asthma.

**Methodology/Principal Findings:**

Corticosteroid sensitivity was determined by the dexamethasone ability to inhibit TNFα-induced IL-8 or LPS-induced TNFα production. PP2A expression, glucocorticoid receptor (GR) nuclear translocation defined as the nuclear/cytoplasmic GR ratio and phosphorylation of GR-Ser^226^, c-Jun N-terminal kinase 1 (JNK1) and PP2A were analysed by Western-blotting. Phosphatase activity was measured by fluorescence-based assay. Okadaic acid (OA), a PP2A inhibitor, reduced corticosteroid sensitivity with reduced GR nuclear translocation and increased GR phosphorylation in U937 monocytic cells. PP2A knockdown by RNA interference showed similar effects. IL-2/IL-4 treatment to U937 reduced corticosteroid sensitivity, and PP2A expression/activity. In peripheral blood mononuclear cells (PBMCs) from severe asthma, the PP2A expression and activity were significantly reduced with concomitant enhancement of PP2A_C_-Tyr^307^ phosphorylation compared with those in healthy volunteers. As the results, GR-Ser^226^ and JNK1 phosphorylation were increased. The expression and activity of PP2A were negatively correlated with phosphorylation levels of GR-Ser^226^. Furthermore, co-immunoprecipitation assay in U937 cells revealed that PP2A associated with GR and JNK1 and IL-2/IL-4 exposure caused dissociation of each molecule. Lastly, PP2A overexpression increased corticosteroid sensitivity in U937 cells.

**Conclusions/Significance:**

PP2A regulates GR nuclear translocation and corticosteroid sensitivity possibly by dephosphorylation of GR-Ser^226^ via dephosphorylation of upstream JNK1. This novel mechanism will provide new insight for the development of new therapy for severe asthma.

## Introduction

Bronchial asthma has been recognized as a chronic inflammatory disease of the airways with increasing trend of its prevalence. Currently, most patients with asthma are well controlled on regular use of inhaled corticosteroid (ICS) with or without long-acting β_2_-agonists (LABAs) [1]. However, small population (approximately 5–10%) of asthmatics develops severe asthma, and has greater morbidity with corticosteroid insensitive and a disproportionate contribution to health care spending [2]. Therefore, understanding the molecular mechanism of corticosteroid insensitivity may provide clues to improve treatment for patients with severe asthma.

The impairment of corticosteroid responsiveness observed in severe asthma has been induced by decreased glucocorticoid receptor (GR) α expression, increased decoy GR receptor (GRβ), defected ligand binding for GR, reduced GR nuclear translocation and GR/glucocorticoid response elements (GREs) binding [3] as well as HDAC2 reduction. In some asthmatics with corticosteroid insensitivity, nuclear translocation of GR in response to dexamethasone was impaired [4]. Although highly phosphorylated GR by mitogen-activated protein kinase (MAPK) might affect GR nuclear translocation [5], the mechanism for the effect is unclear.

Human GR is known to be phosphorylated at three major sites on its N terminus (Ser^203^, Ser^211^ and Ser^226^) [6]. Although phosphorylation of Ser^203^ and Ser^211^ is required for full GR activity [7]–[9], phosphorylation of Ser^226^ is inhibitory to GR function [10]–[12], suggesting that Ser^226^ phosphorylation could be a biomarker for inactivated GR and involved in reduced nuclear retention of active GR.

Previous studies indicate that c-Jun N-terminal kinase (JNK) is responsible for phosphorylation of Ser^226^ on GR inactivation. Phosphorylation of GR at Ser^226^ by JNK has been shown to inhibit GR transcriptional activation [10] and also regulate GR export from the nucleus [11]. We recently found that the level of GR phosphorylation at Ser^226^ was increased in PBMCs from severe asthma [13]. In addition, some phosphatases such as protein phosphatase 2A (PP2A) and protein phosphatase 5 (PP5) have been reported to modify GR phosphorylation [14]. Interestingly, DeFranco et al. [15] demonstrated that PP2A inhibition by okadaic acid led to inefficient nuclear retention of agonist-bound GR. Further, PP2A may intensify GR action through dephosphorylation of JNK and also regulate GR translocation into nucleus directly [16].

We therefore hypothesized that defect of PP2A impairs steroid effects via failure of dephosphorylation of GR at Ser^226^ and we demonstrated this first time in PBMCs obtained from severe asthmatics.

## Results

### PP2A inhibition induced corticosteroid sensitivity

**Figure 1:**
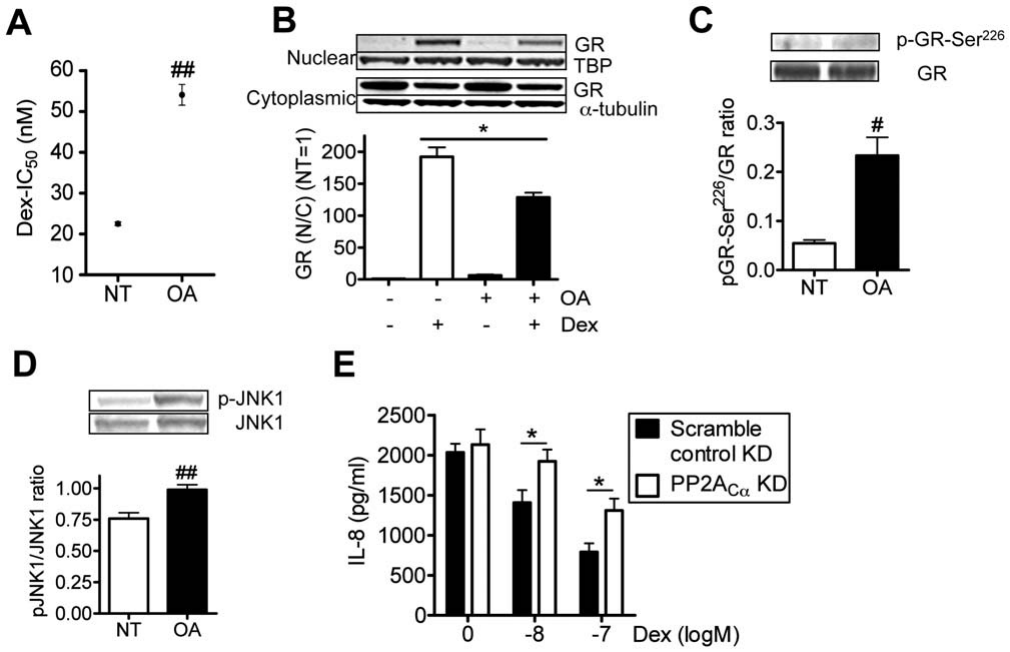
Effects of PP2A inhibitor on glucocorticoid function. Effect of okadaic acid (OA; 10^−9^ M) on corticosteroid sensitivity (A), GR nuclear translocation (B), phosphorylation levels of GR-Ser^226^ (C) and JNK1 (D) in U937 cells (n = 3–4). **E: **Effect of PP2A siRNA on IC_50_ of dexamethasone on TNFα-induced IL-8 (n = 7). Values represent means ± SEM. ^#^ *P*<0.05, ^##^ *P*<0.01 (vs. non-treatment control; NT), * *P*<0.05.

As shown in Figure 1A, pretreatment of okadaic acid (OA; 10^−9^ M) increased IC_50_ values of dexamethasone on TNFα-induced IL-8 release in U937 monocytic cell line by 2.4 fold, suggesting OA reduced dexamethasone sensitivity. OA also significantly inhibited dexamethasone (10^−7^ M)-induced GR nuclear translocation defined as the ratio of nuclear and cytoplasmic GR band density (see Figure 1B). Cell viabilities were more than 90% in all treatments. In addition, OA treatment caused enhanced GR phosphorylation at Ser^226^ and JNK1, which is known upstream kinase of GR phosphorylation (see Figure 1C and D). As OA is not selective PP2A inhibitor, PP2A catalytic subunit α (PP2A_Cα_) has been knocked down by RNA interference. Western blotting analysis confirmed 30% knockdown (KD) of PP2A_C_ in U937 cells and cell viabilities were more than 70% (data not shown). As shown in Figure 1E, PP2A-KD significantly decreased inhibitory effects of dexamethasone on TNFα-induced IL-8 release in U937 cells. Thus, PP2A is a key phosphatase to control corticosteroid function.

### PP2A expression and activity were reduced in IL-2/IL-4 treated corticosteroid insensitive model

**Figure 2:**
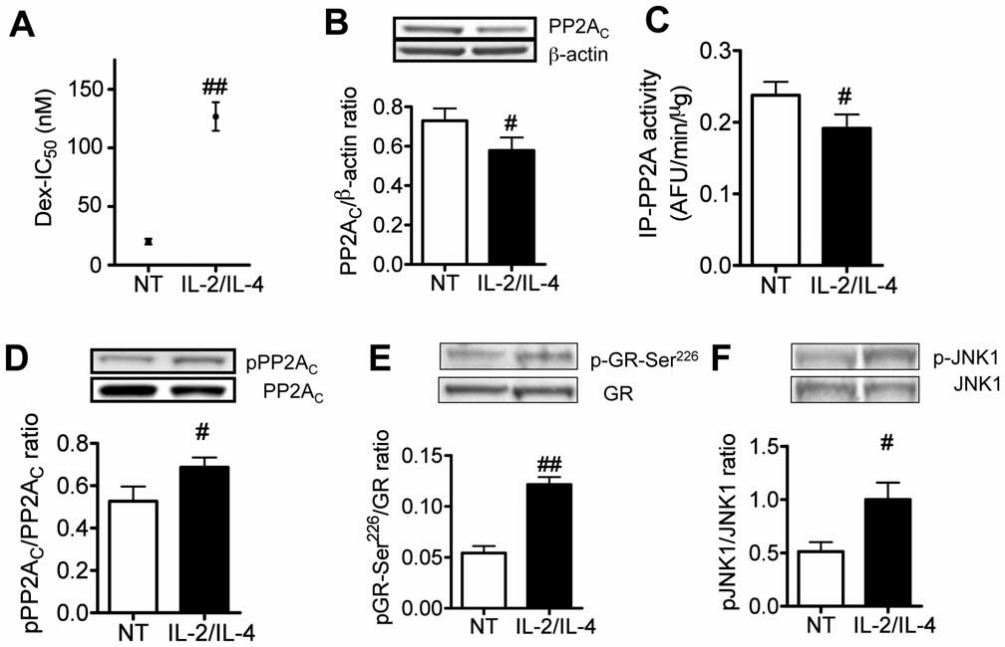
PP2A function in IL-2/IL-4-treated U937 cells. Effects of IL-2/IL-4 co-treatment for 48 h on IC_50_ of dexamethasone on TNFα-induced IL-8 release (A), PP2A_C_ protein expression (B), immunoprecipitated PP2A (IP-P2A) activity (C), PP2A_C_-Tyr^307^ phosphorylation(D), GR-Ser^226^ phosphorylation (E) and JNK1 phosphorylation (F). Values represent means ± SEM (n = 3–4). ^#^ *P*<0.05, ^##^ *P*<0.01 (vs. non-treatment control; NT).

It is well known that co-treatment of IL-2 and IL-4 induces corticosteroid insensitivity in U937 cell. In this experiment, IL-2 (20 ng/ml)/IL-4 (10 ng/ml) treatment for 48 h significantly increased IC_50_ values of dexamethasone on TNFα-induced IL-8 release (see Figure 2A). As seen in Figure 2B, PP2A level in total cell extracts was significantly reduced in IL-2/IL-4 treated cells. Cell viabilities were more than 90% in all treatments. Furthermore, the activity of PP2A immunoprecipitated from cells treated with IL-2/IL-4 was also significantly reduced, suggesting that IL-2/IL-4 reduced both activity and protein expression (see Figure 2C). Although PP2A activity is reported to be reduced when it is phosphorylated at Thr^307^, IL-2/IL-4 significantly increased PP2A phosphorylation. At the same time, IL-2/IL-4 treatment significantly enhanced GR phsophorylation at Ser^226^ and JNK1 (see Figure 2E and F), but not JNK2/3 (data not shown).

### PP2A expression and activity were reduced in PBMCs from severe asthma

**Figure 3:**
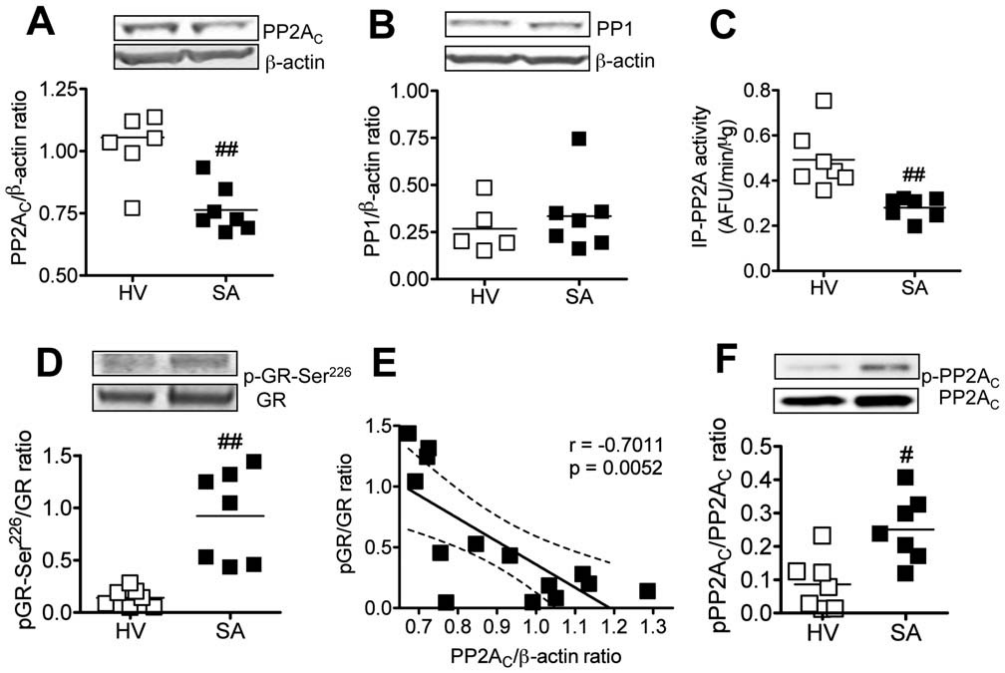
PP2Ac expression and activity in severe asthma. PP2A_C_ protein expression (A), PP1 protein expression (B), immunoprecipitated PP2A (IP-PP2A) activity (C), phosophorylation levels of GR-Ser^226^ (D) and PP2A_C_-Tyr^307^ (F) in PBMCs from severe asthmatics (SA) and healthy volunteers (HV). **E.** Correlation between PP2A_C_ expression and GR-Ser^226^ phosphorylation. The dotted lines show 95% confidence interval. ^#^ *P*<0.05, ^##^ *P*<0.01 (vs. HV).

In PBMCs from patients with severe asthma (SA), protein expression of PP2A, but not PP1, was significantly reduced compared with those from healthy volunteers (HV) (see Figure 3A and B) (PP2A: 1.1±0.06 in HV, 0.76±0.04 in SA). In addition, immunoprecipitated PP2A activity was significantly reduced in PBMCs form severe asthmatics as well as PP2A expression (see Figure 3C *)*. At the same time, phosphorylation levels of GR at Ser^226^ (see Figure 3 D) and JNK1, but not JNK2/3(see Figure S1 A and B), were significantly increased in PBMCs from patients with severe asthma. As shown in Figure 3E, PP2A_C_ protein expression corrected to β actin expression was significantly and negatively correlated with the level of GR-Ser^226^ phosphorylation. PP2A activity was also correlated with GR-Ser^226^ phosphorylation (see Figure S2A). Furthermore, PP2A activity and expression were also negatively correlated with JNK1 phosphorylation (see Figure S2 B and C). There was also a positive correlation between JNK1 and GR-Ser^226^ phosphorylation (see Figure S1C). As same with IL-2/IL-4 model, PP2A was also significantly phosphorylated at Thr^307^ in PBMCs of severe asthma (see Figure 3F).

### PP2A dissociates from GR and JNK1 in steroid insensitive U937 cell model

**Figure 4:**
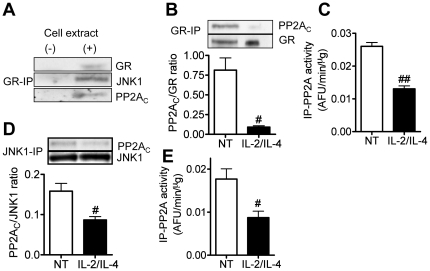
Association between PP2A and GR/JNK1 in U937 cells. (A) PP2A_C_ and JNK1 expression in GR-immunoprecipitates. Expression levels of PP2A_C_ in GR (B)- or JNK1 (D)-immunoprecipitates. PP2A activity in GR (C)- and JNK1 (E) immunoprecipitates were also determined. Values represent means of four experiments ± SEM. ^#^ *P*<0.05, ^##^ *P*<0.01 (vs. non-treatment control; NT).

Whole protein was extracted from non-treated U937 cells, and PP2A and JNK1 were co-immunoprecipitaed with GR. As shown in Figure 4A, PP2A and JNK1 were detected in GR immunoprecipitates, suggesting that GR, PP2A and JNK1 were in same complex. In the same way, co-immunoprecipitation with GR or JNK1 was conducted at 48 h after IL-2/IL-4 treatment. As shown in Figure 4B and 4C, IL-2/IL-4 treatment inhibited PP2A_C_ association with GR, and GR-associated PP2A activity. Furthermore, IL-2/IL-4 treatment also inhibited association of JNK1 and PP2A_C_ (see Figure 4C), and JNK1 associated PP2A activity (see Figure 4E).

### PP2A overexpression increased GR nuclear translocation and increased dexamethasone sensitivity

**Figure 5:**
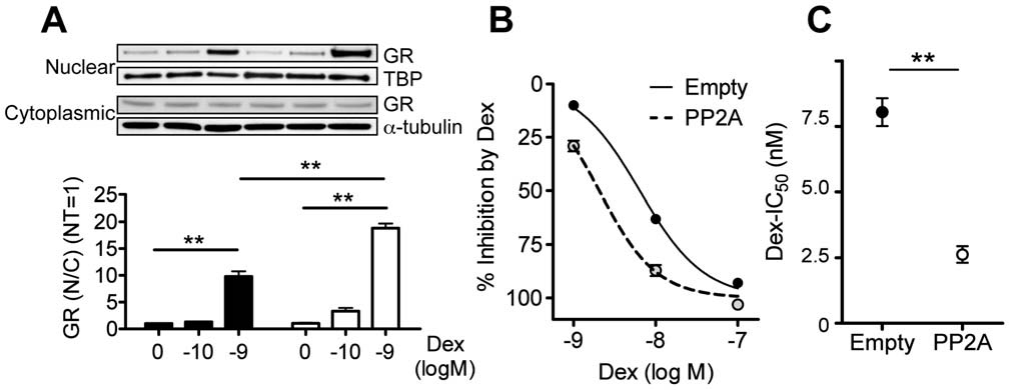
Effect of PP2A overexpression on glucocorticoid function in U937 cells. (A) Fold changes of GR nuclear translocation defined as the ratio of nuclear and cytoplasmic GR (N/C) over no Dex control (NT). Dexamethasone inhibitory response curve (B) and IC_50_ values of Dex (C) on LPS-induced TNFα production. Values represent means ± SEM (n = 3): ** *P*<0.01 between groups.

PP2A overexpression plasmid was transfected and the cells were used 20 h after transfection. Empty vector was also transfected as control. Although dexamethasone at 10^−9^ M significantly increased GR nuclear translocation, PP2A overexpression significantly increased GR nuclear translocation in U937 cells (see Figure 5A). The inhibitory response curve of dexamethasone on LPS-induced IL-8 release was shifted leftward by PP2A overexpression (see Figure 5B), and therefore the IC_50_ value of dexamethasone on IL-8 release was reduced by PP2A overexpression compared with empty vector transfection, suggesting PP2A overexpression increased corticosteroid sensitivity (IC_50_ of dexamethasone: 2.1 nM in PP2A overexpression vs. 6.4 nM in empty plasmid transfected control) (see Figure 5C).

## Discussion

It is previously demonstrated that PBMCs (mainly monocytes) obtained from patients with severe asthma was corticosteroid insensitive [4], [13]. The impairment of corticosteroid responsiveness has been reported to be induced by several mechanisms [3]. Although an increase in decoy receptor, GRβ, has been identified as one of important causes of corticosteroid insensitivity, the GRβ isoform was rarely found in monocyte or U937 cells unlikely in neutrophils and lymphocytes. As another possibility, phosphorylation of Ser^226^ on GRα is reported to be one of major inhibitory mechanisms of corticosteroid function [10]–[12]. In this manuscript, we demonstrated that the level of GR-Ser^226^ phosphorylation was significantly increased in PBMCs in severe asthma compared with those in healthy volunteers in agreement with our previous findings [13]. JNK1 is known to phosphorylate GR-Ser^226^ [10], [11] and phosphorylation level of JNK was increased in PBMC from patients with corticosteroid-resistant asthma [17]. In our samples, JNK1 phosphorylation was also upregulated in PBMCs in severe asthma (Fig. S1A) and there was a good correlation between GR-Ser^226^ phosphorylation and JNK1 phosphorylation (Fig. S1C). Generally the increased phosphorylation level was considered due to highly activated relevant upstream kinase or impaired phosphatase activity. As IL-2/IL-4 co-treatment, a well-known steroid insensitive model [18], [19], showed GR-Ser^226^ phosphorylation and JNK1 activation as well as steroid insensitivity, we used IL-2/IL-4 model for mechanism assay.

Okadaic acid is a PP2A and PP1 inhibitor at around 1 µM, but OA selectively inhibits PP2A at lower concentration (around 1 nM) [20], [21]. PP2A is a serine/threonine phosphatase, which regulates cell signal transduction and many cellular functions [22]. In U937 cells, this low concentration (1 nM) of OA clearly reduced GR nuclear translocation and corticosteroid sensitivity (Fig. 1A and B). PP2A KD by RNA interference also reduced dexamethasone sensitivity (Fig. 1E), but PP1 RNAi did not induce corticosteroid insensitivity (data not shown). Furthermore, there was no significant reduction of PP1 protein expression in PBMCs from severe asthma but PP2A_C_ expression was significantly reduced in severe asthma (Fig. 3A and B). Taken together, PP2A, but not PP1, is likely a key molecule for corticosteroid function.

PP2A has been reported to be involved in GR phosphorylation and GR nuclear translocation [15], [16]. Although it has not been elucidated which site of GR is dephosphorylated by PP2A on regulation of GR nuclear translocation, we demonstrated here that PP2A regulates corticosteroid sensitivity through GR-Ser^226^ phosphorylation and dephosphorylation in U937 cells. Furthermore, we reported first time that PP2A was down-regulated in PBMCs from severe asthmatics with a concomitant increase in GR-Ser^226^ phosphorylation (Fig. 3A, C and D). PP2A protein expression was reduced in PBMCs from severe asthma and IL-2/IL-4 treated U937 cells, and also very interestingly, immunoprecipitated PP2A activity corrected by protein expression was also decreased in both samples. This suggests that the activity itself was decreased as well as total protein level.

PP2A catalytic subunit (PP2A_C_) plays an important role for the regulation of PP2A complexes and activity [23]. PP2A_C_ can be phosphorylated at Tyr^307^ via epidermal growth factor receptor, insulin receptor and tyrosine kinases such as p60^V-Src^ and p56^Lck^ [24], and phosphorylation of PP2A_C_ leads to reduction of its activity [25]. We found that phosphorylation levels of PP2A_C_-Tyr^307^ were significantly increased in PBMCs from severe asthma and IL-2/IL-4 treated U937 cells (Fig. 2D and 3F). These findings suggest that hyperphosphorylated PP2A_C_-Tyr^307^ might be one of mechanisms of PP2A inactivation under steroid resistant condition including severe asthma. The mechanism of the reduction of PP2A protein level was not clear.

As well as PP2A KD study, we also conducted PP2A overexpression by plasmid transfection. Transfection increased PP2A protein level by approximately 1.5 fold in U937 cells (data not shown). PP2A overexpression clearly increased GR nuclear translocation and dexamethasone sensitivity on LPS-induced IL-8 release (Fig. 5). This is another confirmation that PP2A is involved in GR function and also important evidence that PP2A activator can be a novel therapeutic approach for severe asthma.

Protein phosphatase 5 (PP5), one of serine/threonine phosphatases, which associates with GR-heat shock protein 90 complex [26], [27] has also been reported to influence GR actions [28] and dephosphorylate GR-Ser^226^ [14]. Thus involvement of another phosphatase cannot be ruled out.

Thus, we demonstrated that PP2A reduced in PBMCs from patients with severe asthma, and impaired PP2A failed to dephosphorylate GR-Ser^226^ and JNK1. This is one of the molecular mechanisms of corticosteroid insensitivity in severe asthma and possibly for other corticosteroid refractory diseases, and will be a novel therapeutic target for the treatment.

## Materials and Methods

### Reagents

3-(4,5-dimethylthiazol-2yr)-2-5-diphenyltetrazolium bromide (MTT), dimethyl sulfoxide (DMSO), phorbol 12-myristate 13-acetate (PMA) and lipopolysaccharide (LPS) were purchased from Sigma-Aldrich (Poole, UK). Recombinant Human IL-2 and IL-4 were purchased from R&D Systems Europe (Abingdon, UK). As a pharmacological inhibitor, okadaic acid (Calbiochem, Darmstadt, Germany) was used as needed. The rabbit monoclonal antibody to PP2A catalytic subunit (PP2A_C_), the rabbit polyclonal anti-GR (phospho S^226^) antibody, and the mouse monoclonal antibody to β-actin and TATA binding protein TBP were obtained from Abcam (Cambridge, UK). The rabbit polyclonal anti-GR antibody, the mouse monoclonal antibody to PP1, PP2A_C_ (phospho Try^307^) and α-tubulin were obtained from Santa Cruz Biotechnology (Heidelberg, Germany). The rabbit polyclonal anti-phospho-SAPK/JNK and anti-SAPK/JNK antibodies were obtained from Cell Signaling Technology (Danvers, MA). As immunoprecipitation reagents, TrueBlot® anti-rabbit and goat Ig IP beads were purchased from eBioscience (Hatfield, UK).

### Subjects

Peripheral blood mononuclear cells (PBMCs) were obtained from 7 patients with severe asthma and 10 age-matched healthy volunteers, and separated by AccuSPIN (Sigma–Aldrich). The characteristics of subjects were shown in Table 1. This study was approved by the local ethics committee of Royal Brompton and Harefield NHS Trust and written informed consent was obtained from each patient or volunteer.

**Table 1:** Characteristics of subjects.

	Healthy volunteers(n = 10)	Severe asthmatics(n = 7)
Gender (M∶F)	2∶8	3∶4
Age	50.2±2.6	45.7±5.3
Atopic	2/10	5/7
FEV_1.0_ %pred.	92.0±2.4	76.0±5.6*
FEV_1.0_/FVC	78.2±1.5	69.0±3.3*
ICS	none	7/7629±92 µgFP equivalent dose
Oral prednisolone	none	6/717.5±1.7 mg
Albuterol	none	7/7

*p<0.05 (vs. healthy volunteers).

ICS: inhaled corticosteroid, FP: fluticasone propionate.

### Cells

The human monocytic cell line U937 [29] (CRL-1593.2™) was obtained from the American Type Culture Collection (ATCC, Rockville, MD). Cells were cultured in complete growth medium (PRMI 1640; Sigma–Aldrich) supplemented with 10% fetal bovine serum (FBS) and 1% L-glutamine at 37°C in a humidified atmosphere with 5% CO_2_. Cell viability was assessed microscopically by trypan blue staining. Cell toxicity was determined by MTT assay as needed. Cells were exposed to IL-2 (20 ng/ml) and IL-4 (10 ng/ml) for 48 h to induce corticosteroid insensitivity.

### Cell Lysis, Immunoprecipiation, and Western Blotting

Cell protein extracts were prepared using modified RIPA buffer (50 mM Tris HCL pH 7.4, 0.5–1.0% NP-40, 0.25% Na-deoxycholate, 150 mM NaCl with freshly added complete protease (Roche, Mannheim, Germany)), as described previously [30], [31]. Phosphatase inhibitor (Active Motif, Rixensart, Belgium) was used as needed. Nuclear extraction was performed using Active Motif kit. Protein concentration was determined using the Bio-Rad Protein Assay (Bio-Rad). Immunoprecipitation was conducted with anti-PP2A antibody (Bethyl, Montgomery, TX) for phosphatase activity assay or anti-GR Ab or anti-JNK1 antibody (Santa Cruz Biotechnology) for co-immunoprecipitation assay. Protein extracts (40 µg protein per well, and 60 µg protein for phosphoprotein detection) or immunoprecipitates were analyzed by SDS-PAGE (Invitrogen, Paisley, UK) and detected with Western blot analysis by chemiluminescence (ECL Plus; GE Healthcare, Chalfont St. Giles, UK). Protein expression levels of PP2A_C_ and PP1 were expressed relative to β-actin expression used as a control for protein loading. Phosphorylation levels of GR-Ser^226^, JNK1 and PP2A_C_-Tyr^307^ were expressed relative to total GR, JNK1 and PP2A_C_, respectively. In co-immunoprecipitation study, expression PP2A_C_ in GR- or JNK1-immunoprecipitates was expressed relative to GR or JNK1 expression in each immunoprecipitates, respectively.

### Glucocorticoid receptor nuclear translocation

Cells were treated with Dex (10^−7^ M) for 1 h. Nuclear and cytoplasmic GR were measured by Western blot. TBP (for nuclear protein) or α tubulin (for cytoplasmic protein) expression was used as a control for protein loading. As an index of GR nuclear translocation, the ratio of nuclear GR to cytoplasmic GR was calculated.

### Corticosteroid sensitivity

Cells were treated with dexamethasone (Dex) for 45 min, followed by TNFα-stimulation (10 ng/ml overnight) or LPS (100 ng/ml for 4 h). TNFα-induced IL-8 or LPS-induced TNFα concentrations were determined by sandwich ELISA according to the manufacturer's instructions (R&D Systems Europe). IC_50_ values for dexamethasone on IL-8 or TNFα production were calculated using the computer program Prism 4.0 (GraphPad Software Inc., San Diego, CA) as a marker for steroid sensitivity (Dex-IC_50_).

### Protein phosphatase activity

Phosphatase activity was assayed by using the SensoLyteTM MFP Protein Phosphatase Assay system (AnaSpec, San Jose, CA). Cell lysates were immunoprecipitated with anti-PP2A antibody. Immunoprecipitated PP2A with assay buffer (40 mM Tris HCl pH 8.4, 34 mM MgCl_2_, 4 mM EDTA, 4 mM DTT) was transferred to a 96-well plate, and then the same volume of 3-O-methylfluorescein phosphate (MFP) reaction solution in 1 M DTT in assay buffer) was added. PP2A-induced dephosphorylation was monitored by measuring the fluorescence of MFP product. Phosphatase activity was calculated as the slope of fluorescence recordings and expressed as arbitrary fluorescence units per microgram of protein.

### RNA interference

PP2A catalytic subunit α siRNAs (Hs_PPP2CA_6, 5, 7 and 3) and non-silencing scrambled control siRNA (AllStars Negative Control siRNA) were purchased from QIAGEN (Crawley, UK). The siRNA sequences were transfected using an HVJ Envelope (HVJ-E) Vector Kit GenomONE-Neo (Ishikawa Sangyo Kaisha Ltd., Osaka, Japan) by modified methods described in the manufacturer's instructions and by Tsuchiya et al. [32]. Briefly, HVJ-E (0.5 AU) was mixed with 20 µl of siRNA solution (0.5 µM) and 4 µl of Reagent B. After centrifugation, the pellet was resuspended in 25 µl of buffer, followed by the addition of 10 µl of Reagent C. The siRNA-HVJ-E mixture was combined with 1×10^6^ cells and centrifuged at 12,000 rpm for 20 min at 4°C. The cells were resuspended in 2 ml of culture media and incubated for 72 h.

### Transfection

Transfections were done by Nucleofection® (Lonza, Basel, Switzerland), according to the manufacturer's instructions. 2 µg of DNA/plasmids (pCMV6 Entry, OriGene Technologies, Rockville, MD) containing the human PP2A catalytic subunit, alpha isoform (PP2A_Cα_) gene were transfected to U937 cells pretreated with 50 ng/ml of PMA for 4 h. 20 h after the transfection, the medium was changed to the appropriate treatment in 1% FBS medium.

### Statistical analysis

Comparisons of two groups of data were performed using Mann-Whitney U test or paired t test. Correlation coefficients were calculated with the use of Pearson method or Spearman's rank method. Other data were analyzed by ANOVA with post *hoc* test adjusted for multiple comparisons (Bonferroni's test), as appropriate. The difference was considered statistically significant if *P*<.05. Descriptive statistics were expressed as the mean ± SEM.

## Supporting Information

Figure S1 **JNK phosphorylation levels in PBMCs from severe asthmatics.** Phosphorylation levels of JNK1 (A) and JNK2/3 (B). **C,** Correlation between JNK1 phosphorylation and GR-Ser^226^ phosphorylation levels (seven healthy volunteers; HV and seven severe asthmatics; SA). The dotted lines show 95% confidence interval. Individual values and means of seven subjects are shown: ^##^ *P*<0.01 (vs. HV).
(TIF)

Figure S2 **Correlation between PP2A and GR-Ser^226^/JNK1 phosphorylation.** A and B, Correlation between immunoprecipitate PP2A (IP-PP2A) activity and GR-Ser^226^ (A)/JNK1 (C) phosphorylation levels (four healthy volunteers; HV and seven severe asthmatics; SA). C, Correlation between PP2A_C_ protein expression and JNK1 phosphorylation levels (seven HV and seven SA). The dotted lines show 95% confidence interval.
(TIF)
