# Supplementary figures and images for: Defects of Protein Phosphatase 2A Causes Corticosteroid Insensitivity in Severe Asthma
Source: PLoS One. 2011 Dec 19;6(12):e27627. doi: 10.1371/journal.pone.0027627 (PMC3242752; doi:10.1371/journal.pone.0027627)

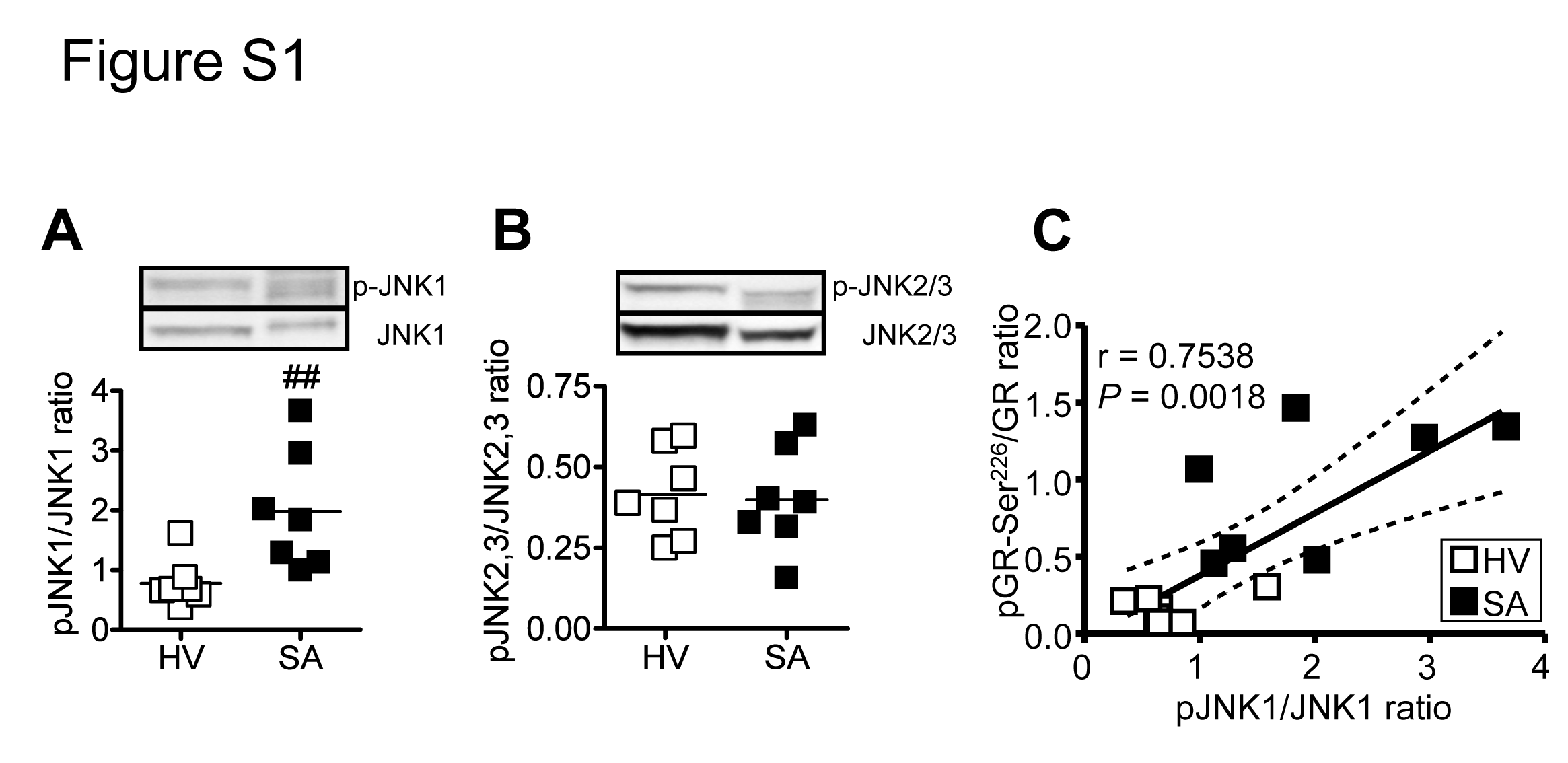

Supplement: Figure S1 — JNK phosphorylation levels in PBMCs from severe asthmatics. Phosphorylation levels of JNK1 (A) and JNK2/3 (B). C, Correlation between JNK1 phosphorylation and GR-Ser226 phosphorylation levels (seven healthy volunteers; HV and seven severe asthmatics; SA). The dotted lines show 95% confidence interval. Individual values and means of seven subjects are shown: ## P<0.01 (vs. HV). (TIF) [file pone.0027627.s001.tif]

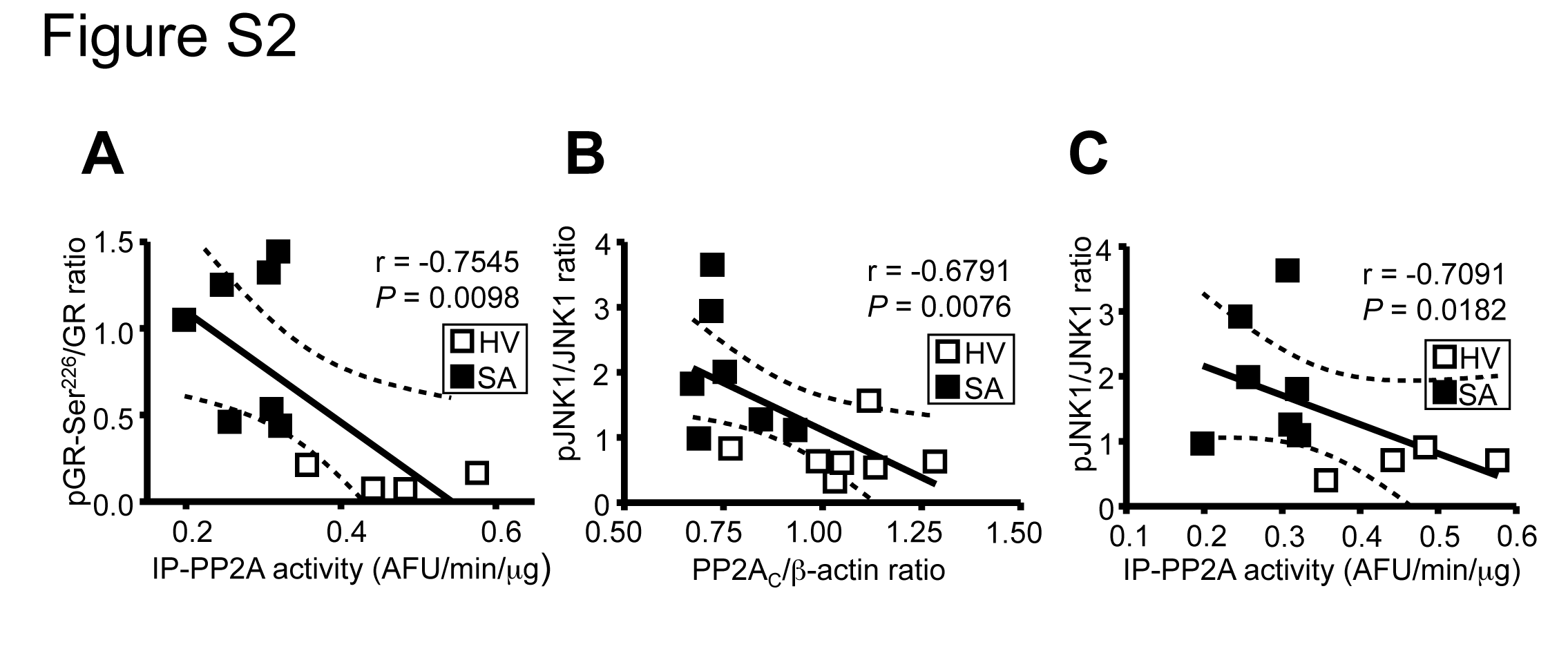

Supplement: Figure S2 — Correlation between PP2A and GR-Ser226/JNK1 phosphorylation. A and B, Correlation between immunoprecipitate PP2A (IP-PP2A) activity and GR-Ser226 (A)/JNK1 (C) phosphorylation levels (four healthy volunteers; HV and seven severe asthmatics; SA). C, Correlation between PP2AC protein expression and JNK1 phosphorylation levels (seven HV and seven SA). The dotted lines show 95% confidence interval. (TIF) [file pone.0027627.s002.tif]
